# Evaluation and modification of lanthanum-based flocculation for isolation of bacteria from water samples

**DOI:** 10.1016/j.btre.2018.e00267

**Published:** 2018-06-19

**Authors:** Linda Jansson, Ronnie Eriksson, Johannes Hedman, Moa Lavander

**Affiliations:** a Applied Microbiology, Lund University, SE-221 00 Lund, Sweden; b Swedish National Forensic Centre, SE-581 94, Linköping, Sweden; c Science Division, Biology Department, National Food Agency, SE-753 19, Uppsala, Sweden

## Abstract

Molecular detection of pathogenic microorganisms in drinking and natural water is often challenged by low concentrations of the sought-after agents. Convenient methods to concentrate bacteria from water samples ranging from 1-10 L are highly warranted. Here we account for the evaluation of a lanthanum-based flocculation method to concentrate bacteria from water samples, applying four different bacterial species in tap water as well as river water. Our results show that the success of lanthanum-based flocculation is determined by both the bacterial species and the nature of the water sample. For tap water, satisfying flocculation efficiencies (above 60 %) were only reached for autoclaved water samples. However, the performance of the lanthanum-based flocculation method for non-autoclaved water was markedly improved by the addition of 20 mM bicarbonate to increase alkalinity. Our modified flocculation protocol may be applied as an alternative concentration method for bacteria in water samples of one liter or more.

## Highlights

• A published lanthanum-based flocculation protocol is evaluated for four bacterial species
• The success of lanthanum-based flocculation is determined by both the bacterial species and the nature of the water sample
• Addition of 20 mM bicarbonate significantly improve the flocculation efficiency for tap water

## 1 Introduction

Molecular detection of pathogenic microorganisms in drinking and natural water is often challenged by low concentrations of the sought-after agents. In general, volume reduction of the water samples is needed to reach satisfying limits of detection. For larger volumes of water (>10 L), ultrafiltration methods are suitable and efficient [[1], [2], [3]], while bacteria in volumes below one liter can be concentrated by centrifugation or membrane filtration. However, convenient methods to concentrate bacteria from water samples ranging from 1-10 L are highly warranted. Here we account for the evaluation of a published lanthanum-based flocculation method [4] to concentrate bacteria from water samples of one liter. We also present a promising modification to the protocol. The flocculation protocol is completed within hours and requires little manual input. It has previously been shown to give *E. coli* DNA recovery rates of 81-111 % and 61-105 % for tap water and autoclaved raw water, respectively [4]. These published results are very promising, but for lanthanum-based flocculation to be broadly applied, the method must be validated for other bacteria and waters. We evaluated the method for flocculation and detection of *Bacillus cereus* (F2085) [5], *Salmonella enterica* serovar Typhimurium *(*CCUG-98112-08), *Escherichia coli* (DH5-alpha), and the Live Vaccine Strain of *Francisella tularensis* ssp holarctica (NC_007880), spiked in different numbers ranging from 10^3^-10^7^ CFU to one liter of autoclaved and non-autoclaved tap water. Non-autoclaved river water (Höje river, Lund, Sweden) was tested in subsequent experiments.

## 2 Materials and methods

Lanthanum(III) chloride (262072, Sigma-Aldrich) was added to the water samples to a final concentration of 0.2 mM [6], and the original bacterial flocculation protocol in [4] was pursued. The modified protocol included addition of 20 mM bicarbonate to the water samples prior to adding the lanthanum(III) chloride. After 20 minutes of mixing and one hour sedimentation, the supernatants (900 mL) were removed carefully without disturbing the settled flocs. Samples of one mL were taken from the supernatant and the remaining homogenized floc phase respectively.

For DNA extraction, bacteria were pelleted by centrifugation at 5000 × g for 5 minutes. The supernatants were removed and pellets resuspended by vortexing in 175 μL lysis buffer G2 (EZ1 DNA Tissue kit) with 200 μg Endoproteinase K (P2308, Sigma-Aldrich) and 50 μg RNase A (EN0531, Thermo Scientific). Samples were incubated in water bath at 56 °C for 40 minutes. After this pre-lysis step, the samples were further extracted in an EZ1 extraction robot using EZ1 DNA Tissue Kit (953034, Qiagen) and EZ1 DNA Bacterial Card (9016362, Qiagen), elution volume 50 μL. Samples were analyzed in duplicates with qPCR, for *B. cereus* and *Salmonella* on an ABI 7300 instrument (Applied Biosystems) and for *E. coli* and *F. tularensis* on a LightCycler Nano instrument (Roche Diagnostics). The reaction mix included 1 × Immobuffer (Bioline), 0.2 mM nucleotides (Roche Diagnostics), 4 or 2.5 mM MgCl_2_ when probes or EVAGreen was used respectively (Roche Diagnostics), 1 × ROX Reference Dye (ThermoFisher Scientific) when using the ABI 7300, 2 μg of BSA (Roche Diagnostics), 1 U Immolase DNA polymerase (Bioline), assay-specific primers and probes or EVAGreen according to below, 2 μL sample and SuperQ water up to 25 μL for the ABI 7300 and to 20 μL for the LightCycler Nano. Primers and probes were applied as follows: 0.3 μM invA primers and 0.2 μM invA probe for detection of *Salmonella* [7], 0.5 μM A_bacillus primers and 0.1 A_bacillus probe for detection of *B. cereus* [8], 0.3 μM iQFt-1 primers and 1x EVAGreen for detection of *F. tularensis* [9], and 0.3 μM uidA primers and 1× EVAGreen for detection of *E. coli* [10]. PCR conditions for *B. cereus* and *Salmonella* were 10 minutes at 95 °C followed by 45 cycles of 15 s at 95 °C and 50 s at 60 °C. For *E. coli* and *F. tularensis*, 10 minutes at 95 °C were followed by 45 cycles of 10 s at 95 °C and 30 s at 60 °C and a melt curve analysis from 60 °C to 97 °C with a temperature increase of 0.1 °C/s.

For quantification, a standard curve for each species was constructed from a tenfold dilution series ranging from 10°-10^-5^ ng/μL of DNA (using the EZ1 protocol described above) from overnight bacterial cultures. The extracts were quantified using Qubit dsDNA BR assay kit on a Qubit 3.0 instrument (ThermoFisher Scientific). Statistical analysis was performed using Student’s t test and Pearson’s correlation coefficient.

The flocculation efficiency was calculated according to:

$$Flocculation\;\;efficiency\;(\%)=\left(\frac{C_{floc}\;\;x\;\;V_{floc}}{C_{floc}\;\;x\;\;V_{floc}+C_{supernatant\;}\;x\;{\;V}_{supernatant}}\right)x100$$

where C_floc_ and V_floc_ are the bacterial DNA concentration and total volume of the floc phase, and C_supernatant_ and V_supernatant_ are the bacterial DNA concentration and total volume of the supernatant phase [4]. Since the floc phase and the supernatant were approximately 100 mL and 900 mL respectively, flocculation efficiencies close to 10 % indicate failed flocculation, i.e. no bacteria were associated with the settled flocs.

## 3 Results and discussion

**Fig. 1:**
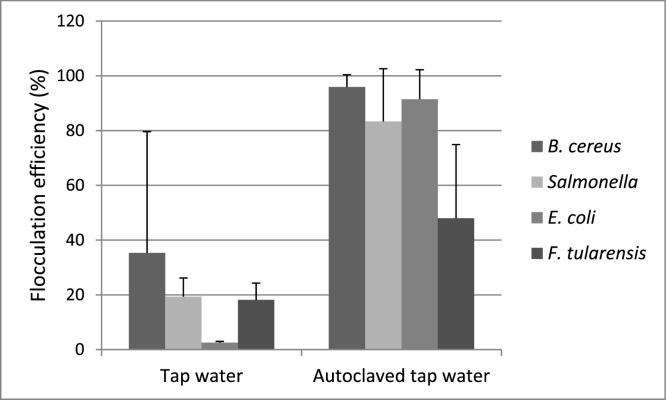
Flocculation efficiencies for *B. cereus*, *Salmonella, E.coli* and *F. tularensis* in non-autoclaved tap water (n = 3 - 6) and in autoclaved tap water (n = 2 - 16). Data are presented as mean values and standard deviations.

Applying the published protocol, we noticed considerably higher flocculation efficiencies for autoclaved tap water compared with non-autoclaved tap water (Fig. 1). Satisfying flocculation efficiencies (above 60%) were reached for three of the four bacteria in autoclaved tap water, but for no bacteria in non-autoclaved tap water. With increased temperature and pressure during autoclaving, the solubility equilibrium in the water is shifted and the ionic strength and alkalinity increase. This may facilitate the flocculation process, as it is known that addition of e.g. bicarbonate increases alkalinity and also the rate of flocculation reactions [11].

Thus, in an attempt to improve lanthanum-based flocculation efficiency for non-autoclaved water, we added bicarbonate (NaHCO_3_) in concentrations from 10 to 40 mM, and adjusted the pH to 7, 8 or 9. Adjusting the pH had no noticeable effect, but addition of 10 or 20 mM bicarbonate increased the flocculation efficiency significantly (data not shown). Our modified protocol thus included the addition of 20 mM bicarbonate. To assess the effect of initial bacterial concentration on flocculation efficiency, we added 10^3^-10^7^ CFU of each bacterial species to one liter non-autoclaved tap water, comparing the original protocol [4] to the modified protocol. The modified method gave significantly higher flocculation efficiencies for all bacterial species (p values from Student’s t-test ≤0.005, Table 1). No correlation between cell concentration and flocculation efficiency was seen; Pearson’s correlation coefficient was determined to 0.061, p = 0.76 for the original protocol, and to 0.22, p = 0.24 for the modified protocol. For some low concentration samples, the flocculation efficiency could not be determined due to negative qPCR results for either supernatant or floc. However, positive detection was more frequent in the flocs compared with the supernatants (Table 1). Further, we evaluated our modified protocol for non-autoclaved river water (Höje river, Lund, Sweden). *B. cereus*, *Salmonella* and *F. tularensis* were added at 10^6^ CFU per species to one liter water samples. No improvements in flocculation efficiencies were seen for the modified protocol (*B. cereus* 80 % (modified) compared to 77 % (original), *Salmonella* 20 % (modified) compared to 29% (original), *F. tularensis* 23 % (modified) compared to 19 % (original)).

**Table 1:** Flocculation efficiencies for four different bacterial species in five different initial concentrations. Comparisons are made between the original method [4] and the modified protocol with addition of 20 mM bicarbonate, applying tap water (non-autoclaved). Data are presented as mean values with standard deviations (n = 2). If no standard deviation is presented, flocculation efficiency could only be determined for one of the replicates. N/A indicates that the flocculation efficiency could not be determined for any of the replicates, due to negative qPCR results for either the supernatant or the floc. When flocculation efficiency is missing for at least one replicate, the number of floc samples resulting in positive qPCR detection is given in brackets.

		Flocculation efficiencies (%) with CFU/liter of 10^7^-10^3^				
Bacterium	Method	10^7^	10^6^	10^5^	10^4^	10^3^
*B. cereus*	Original	5.6 ± 2.6	5.2 ± 1.5	9.2 ± 12.7	3.9 (2/2)	N/A (1/2)
	Modified	98.8 ± 0.2	99.8 ± 0.1	85.5 ± 20.0	91.0 (2/2)	N/A (2/2)

*Salmonella*	Original	10.8 ± 6.8	7.6 ± 0.04	4.7 (1/2)	N/A (0/2)	N/A (0/2)
	Modified	25.9 ± 0.8	48.7 ± 8.1	32.1 (1/2)	N/A (2/2)	N/A (0/2)

*E. coli*	Original	5.4 ± 1.5	2.5 ± 1.9	1.7 ± 1.0	N/A (2/2)	0.7 (1/2)
	Modified	79.5 ± 1.5	94.7 ± 1.4	55.6 ± 35.8	12.0 (2/2)	36.3 (1/2)

*F. tularensis*	Original	5.4 ± 2.1	4.3 ± 3.4	8.8 ± 12.4	9.6 ± 11.5	5.7 (1/2)
	Modified	29.9 ± 9.8	48.3 ± 0.5	20.6 ± 24.1	54.1 ± 32.6	41.3 ± 21.4

## 4 Conclusions

Our results show that the success of lanthanum-based flocculation of bacteria is determined by both the bacterial species and the nature of the water sample. The original method [4] can be markedly improved by adding 20 mM bicarbonate to increase alkalinity. Our modified flocculation protocol may be applied as an alternative concentration method for bacteria in water samples of one liter or more for which centrifugation is laborious.

## Conflict of Interest

None.
